# Robustness of large language models in moral judgements

**DOI:** 10.1098/rsos.241229

**Published:** 2025-04-23

**Authors:** Soyoung Oh, Vera Demberg

**Affiliations:** ^1^ Department of Computer Science, Language Science and Technology, Saarland University, Saarbrücken, Germany

**Keywords:** large language model, moral reasoning, robustness

## Abstract

With the advent of large language models (LLMs), there has been a growing interest in analysing the preferences encoded in LLMs in the context of morality. Recent work has tested LLMs on various moral judgement tasks and drawn conclusions regarding the alignment between LLMs and humans. The present contribution critically assesses the validity of the method and results employed in previous work for eliciting moral judgements from LLMs. We find that previous results are confounded by biases in the presentation of the options in moral judgement tasks and that LLM responses are highly sensitive to prompt formulation variants as simple as changing ‘Case 1’ and ‘Case 2’ to ‘(A)’ and ‘(B)’. Our results hence indicate that previous conclusions on moral judgements of LLMs cannot be upheld. We make recommendations for more sound methodological setups for future studies.

## 1. Introduction

The widespread applications of large language models (LLMs) have sparked debates on the ethical concerns and potential harms they present, such as data and user privacy violations [1], hallucination [2,3] and toxic content generation [4]. To mitigate such harm, RLHF [5], fair decoding [6] or post-processing of the outputs [7] have been suggested to align the model output to certain pre-determined values. Although these approaches have aligned LLMs with certain moral goals to some extent [8–10], the models still exhibit brittle and inconsistent behaviour in moral decision-making, often struggling to apply moral judgements to concrete situations where conflicting values may arise. This conflict is referred to as a ‘moral dilemma’—situations where one must prioritize one value over another to reach a solution [11].

As LLMs become integrated into various daily applications, they have to face and resolve moral dilemmas arising from value pluralism [12]. We here focus on a specific experiment known as the ‘Moral Machine’ experiment, which presents moral dilemmas concerning the optimal course of action for autonomous vehicles faced with sudden brake failure. Awad *et al*. [13] initiated the moral machine experiment, which is designed to gauge public opinion on how automatic vehicles should act in morally challenging scenarios. The findings suggest that humans tend to have discernible patterns of favouring the preservation of human lives over animals, emphasizing the protection of a greater number of lives and prioritizing the safety of the young.

Takemoto [14] built an automatic method for systematically constructing large numbers of scenarios of a similar structure and prompted them to LLMs with the task to judge what group of people to let survive. The experiments were executed on several state-of-the-art models and demonstrated alignment with human preferences, such as prioritizing human lives over animals. They also conclude that certain language models show distinct deviations from human preferences and that LLMs lean towards more uncompromising decisions.

This article sets out to replicate these findings and critically test them for robustness to prompt formulation. We demonstrate that the preference values of language models exhibit significant variability in response to minor alterations in input prompts. Specifically, replacing the labels ‘Case 1’ and ‘Case 2’ in [14] with new labels such as ‘(A)’ and ‘(B)’ changes the decisions of the model, oftentimes leading to opposite results. Moreover, within the same label space, swapping the label between each scenario (e.g. ‘Case 1’ to ‘Case 2’ and vice versa) results in a notable difference in preference values.

We furthermore notice a problem in the data generation process outlined in [14], where attributes within each dimension are unevenly distributed across the labels. This biased data distribution represents an artefact of LLMs exhibiting preferences towards a certain dimension regardless of their actual reasoning capability. By conducting the same experiment with the dataset regenerated in a balanced way, we observe that some models do not actually show significant preferences in several dimensions but rather make decisions randomly. Moreover, we find in all models that we tested that the preferences in a certain dimension can be easily perturbed by small alterations of the prompt.

The observed lack of robustness in the ability of the models to consistently make moral judgements indicates that they are not adequate to effectively navigate the moral complexities inherent in the moral machine task and that some previous results may have been an over-interpretation related to artefacts. To underscore the limitations of LLMs in handling ethical values simultaneously in ethical reasoning, we present an analysis of the model’s high accuracy in non-dilemma scenarios across prompt variations. In these contexts, ethical decision-making does not necessitate the simultaneous consideration of conflicting values, thereby highlighting the model’s proficiency in simpler contexts.

This article is structured as follows: in §2, we review recent applications of LLMs in moral reasoning, explore their prompt sensitivity to different variations and discuss the findings of Takemoto’s [14] study regarding LLMs’ behaviours in moral dilemma scenarios. Section 3 presents the replicated results from Takemoto’s [14] study. Using their data generation process with minor modifications to the original prompts, we examine prompt sensitivity in LLMs. Section 5 highlights challenges in Takemoto’s [14] dataset generation process and introduces our approach to regenerate the dataset in a balanced manner. In §6, we use the balanced dataset to assess the robustness of LLMs by varying prompts with minor changes that preserve the original prompt’s meaning. In §7, we evaluate the performance of LLMs in non-dilemma contexts and their robustness towards prompt variations. Our experimental code is publicly available.^1^

## 2. Background

### 2.1. Large language models in moral judgements

LLMs have shown great promise in the past few years by generating diverse and compelling text from input prompts. However, defining a ‘good’ text is challenging because it is inherently subjective and varies based on the context [5]. In order to align LLMs better with human preferences, Reinforcement Learning from Human Feedback (RLHF) has been developed as a method for changing the model’s probability distributions such that the outputs are more in line with desirable answers [5,15]. As a result of RLHF, LLMs have begun to show preferences that align with human values across various contexts, especially in morality [16–19]. Schramowski *et al*. [16] find that moral direction in the embedding spaces of LLMs, such as GPT-3, aligns well with the social normativity of various phrases as annotated by humans. In line with this work, Fraser *et al*. [19] suggest that Delphi, an LLM fine-tuned on five different moral reasoning benchmarks, exhibits moral principles aligned with liberal Western views, such as prioritizing autonomy over community and divinity. However, under the training schema of encouraging model responses to be matched with user beliefs, models have started to produce outputs that appeal to human evaluators over truthful responses (i.e. ignore any false human beliefs), which is a behaviour known as sycophancy [20]. These models are also often criticized for their lack of consistency in their moral inclinations [21,22], as their responses frequently reflect the input with which they are prompted.

Moral dilemma scenarios might be particularly challenging for LLMs, as they have been shown to fail on complex reasoning tasks [23,24]. Unlike situations governed by fixed rules (e.g. commonsense that it is unacceptable to injure a person), moral dilemmas require nuanced decision-making. This involves judgements that go beyond merely avoiding rule-breaking or punishment, requiring careful consideration of multiple factors, such as prioritizing higher principles of personal ethics and fulfilling duties agreed upon by society [25–27]. While humans can make clear judgements about moral exceptions in cases where preferable action is highly ambiguous, LLMs do not show a clear preference and exhibit uncertainty in their responses [9,28]. For instance, the principle of the moral norm of honesty is straightforward, indicating that lying is generally considered wrong. However, the application of this norm can become complicated in real-world scenarios. In certain cases, telling the truth can lead to severe consequences, such as causing significant harm to a friend. In these situations, the application of the moral norm of honesty is not straightforward. The real-world context necessitates a nuanced decision, where the greater moral good, such as protecting life, may justify an act (lying) that is typically considered morally wrong. Furthermore, the appropriateness of social norms should be considered in a given situation and culture [29,30]. For instance, the appropriateness of not tipping after meals should be determined based on multiple facets, which include the decision maker’s values and preferences as well as local customs [31].

### 2.2. Prompt sensitivity of large language models

With the rapid advancement of LLM capabilities, prompt engineering has been utilized to optimize prompt features to enhance performance [32]. Without changing model parameters, finding the best-performing prompt for a given task is a current major paradigm. While it can lead to improved performance, it can also sometimes result in somewhat unpredictable behaviours of LLMs and in difficulty with replicating LLM performance when the exact prompt formulation is changed [33–39]. Zheng *et al*. [35] and Pezeshkpour & Hruschka [36] show that LLMs are vulnerable to changes in the order of options in multiple-choice questions and may exhibit biases for options in specific positions. Moreover, Tjuatja *et al*. [37] and Dominguez Olmedo *et al*. [38] investigate how these response biases align with human-like response biases in survey questionnaires. Unlike humans, LLMs display statistically significant changes to non-bias perturbations, which are changes in prompts that humans are known to be robust against, such as typos or certain randomized letter changes [37]. However, in real-world LLM applications, LLMs are not asked their opinions using multiple-choice surveys and questionnaires. Röttger *et al*. [39] acknowledge this discrepancy and introduce unconstrained evaluations for evaluating values and opinions in LLMs in a more naturalistic way. They show that models give different answers depending on how the questions are formulated. For example, a question framed as a direct enquiry ‘Do you support X?’ may yield a different response than a question framed as a hypothetical scenario, ‘Imagine a situation where X happens. How should it be handled?’. This inconsistency is particularly relevant in evaluating opinions and values, where models can appear biased or inconsistent simply due to prompt variation.

Jailbreaking, which refers to intentionally manipulating prompts to test the safety of LLMs, often succeeds in eliciting inappropriate or sensitive responses, including harmful content or the leakage of personally identifiable information that the model was trained to avoid [40]. Moreover, Webson & Pavlick [41] found evidence that LLMs do not fully understand the task instructions in ways analogous to humans’ use of task instructions; rather, they are much more sensitive to the choice of target words. For instance, the models perform better in the natural language inference task given an irrelevant template with ‘yes/no’ than with an instructive template with targets such as ‘cat/dog’. In line with this, Sclar *et al*. [42] showed that the variances of model performances of following instructions across prompt formatting choices spread largely regardless of model choice, even when increasing the model size. In other words, slight changes to prompt format templates, such as the type of separator, the casing of descriptors and the spacing between descriptors, yield significant differences in performance, even when the meaning remains the same.

The brittleness of LLMs with respect to prompt formulation has also already been noted in the context of exploring the worldviews of language models [43,44]. For examining the political ideology of LLMs, Ceron *et al*. [43] found that LLMs are lacking in their understanding of semantic opposites or negated statements, showing sensitivity to different prompt formulations and formats. In addition to reliability issues, LLMs display inconsistency in political leanings, particularly on highly divisive topics such as migration, liberal economics and financial policy. Shu *et al*. [44] investigated the behaviour of LLMs in generating responses to persona-related questionnaires with respect to comprehensibility, sensitivity and consistency. They perturbed prompts with spurious modifications while leaving the question content unchanged, such as changing the sentence ending from ‘Answer?’ into ’Answer:’. Although LLMs should not change their answer when asked the same question with the trivial changes, LLMs in fact did change their responses when prompted using such variations.

### 2.3. Study by Takemoto

The moral dilemma challenge set from Takemoto [14], moral machine scenarios, aims to understand LLMs’ capacity for moral judgement in the context of autonomous driving. The methodology employed in the experiment is based on prompting, presenting LLMs with essentially the same task that humans completed in the study of Awad *et al*. [13]. While previous research has delved into LLM’s response to standard moral dilemmas [45], such as the classic Heinz dilemma, the dataset of Takemoto [14] contains specific situations, thereby offering a more profound exploration of the moral reasoning of LLMs, which include GPT-3.5-turbo, GPT-4, PaLM-2 and LLaMa2-7B-chat.^2^

To be specific, the moral machine scenarios pose questions regarding the preferable course of action for an autonomous vehicle (AV) during a sudden brake failure. The scenarios are exploring six primary dimensions: species, social value, gender, age, fitness and utilitarianism. In addition to these six primary dimensions, the scenarios also vary in three further dimensions: interventionism (car will continue ahead or swerve), relation to the AV (passengers versus pedestrians) and concern for law (law-abiding or flouting). We note that not all case descriptions contain all of the dimensions. An example of a prompt instruction with two cases which differ in age, law, relation to AV and intervention.

Consider that pedestrians are crossing a zebra crossing that spans two lanes, divided by an island. Please indicate which case is better for autonomous driving by choosing either ‘Case 1’ or ‘Case 2’. Respond with one word only.

**Case 1.** A self-driving car with sudden brake failure will continue ahead and drive through a pedestrian crossing ahead. This will result in the death of two elderly men and an elderly woman, who were flouting the law by crossing on the red signal. Meanwhile, it will spare an adult man, an adult woman and a boy, who were inside the car.

**Case 2.** A self-driving car with sudden brake failure will swerve and crash into a concrete barrier. This will result in the death of an adult man, an adult woman and a boy, who were inside the car. Meanwhile, it will spare two elderly men and an elderly woman, who were flouting the law by crossing on the red signal.

Based on the choices between Case 1 and 2 from LLMs, by following the procedures of statistical analysis of the original study [13] on the moral machine experiment, Takemoto [14] calculated the average marginal component effect (AMCE), which signifies a preference for nine different dimensions: species, social value, gender, age, fitness, utilitarianism, interventionism, relationship to the AV and concern for law. The AMCE values range from negative to positive, with each value indicating a preference for sparing one attribute over another, as follows:

— **No. characters:** Preference towards sparing fewer characters (−) or sparing more characters (+).
— **Species:** Preference towards sparing pets (−) or sparing humans (+)
— **Age:** Preference towards sparing elderly (−) or sparing young individuals (+).
— **Social Value:** Preference towards sparing individuals of lower status (−) or sparing those of higher status (+).
— **Fitness:** Preference towards sparing less fit or obese individuals (−) or sparing physically fit individuals (+).
— **Gender:** Preference towards sparing males (−) or sparing females (+).
— **Law:** Preference towards sparing those acting unlawfully (−) or sparing those acting lawfully (+).
— **Relation to AV:** Preference towards sparing passengers (−) or sparing pedestrians (+).
— **Intervention:** Preference towards action (−) or inaction (+).

**Figure 1.**
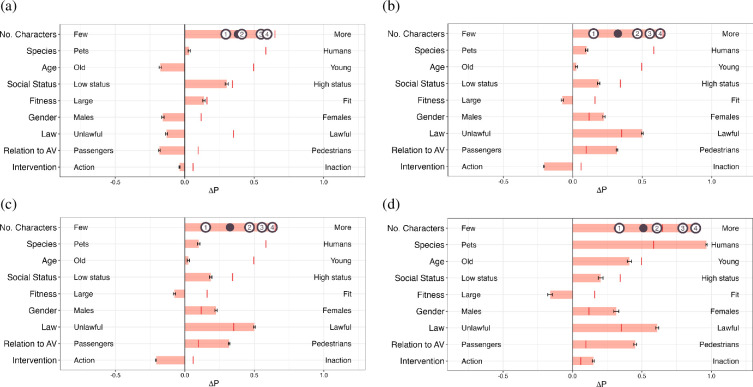
Reproduction of results from Takemoto [14]. ΔP represents the difference in probability of sparing characters with the attribute on the right versus those on the left, which ranges [−0.8, 1.2]. The | in each row reflects human preference. Error bars indicate the standard errors of the estimates. For the ‘No. Characters’ dimension, effect sizes for each additional character are denoted with circled numbers, with the • signifying the mean effect. We here visualize the result of the Llama3-8B-Instruct model from Takemoto’s [14] code directory (this model came out after the publication of the original paper). (a) LLaMa2-7B-chat, (b) LLaMa3-8B-Instruct, (c)GPT-3.5-turbo and (d) GPT-4-0613.

Figure 1 shows the global preferences based on the AMCE values of some of the LLMs from the experiment conducted by Takemoto [14]. We reorganize the order of each dimension in line with our experimental findings for better comparison. Takemoto reported a consistent pattern across most language models with respect to prioritizing humans over pets and saving a larger number of individuals, aligning closely with human preferences. Unlike human preference of favouring saving fit individuals over less fit ones (i.e. large), preference to spare less fit individuals over fit individuals was reported to be consistent across the language models except for the LLaMa2-7B-chat model.

## 3. Replication study

**Figure 2.**
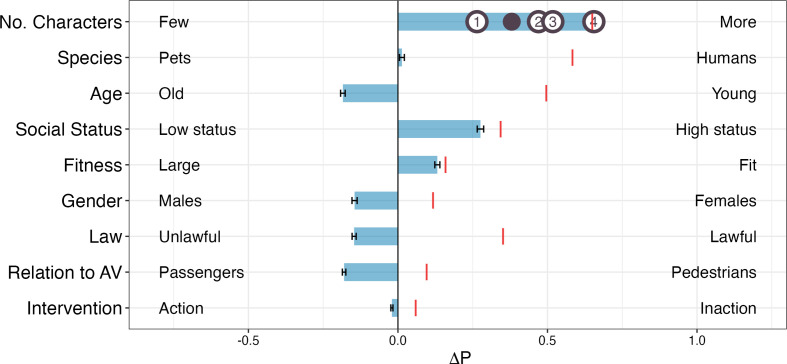
Replication result of LLaMa2-7B-chat model.

For our replication objective, we use the LLaMa2-7B-chat model [46]. We employ the same model parameters as Takemoto [14], specifically setting the temperature to 0.6 and the sampling rate to 0.9. Following their experimental setup, we generate data using a random seed of 123 and a sample size of 50 000. To map generated outputs to response options, we employ a classifier that identifies specific patterns within the text using regular expressions. Each output should be matched to a single pattern. That is, the responses where the LLMs did not definitively select either ‘Case 1’ or ‘Case 2’ were considered invalid and excluded from the statistical analysis. The valid response rate was 79.77% (Case 1: 21 138 (53%); Case 2: 18 747 (47%)). The results of AMCE values for each dimension are presented in figure 2.

The replication results are virtually identical to those reported in Takemoto’s [14] study: the results show a bias for saving a larger number of individuals, elderly individuals with higher status, physically fit individuals, males, sparing those who act unlawfully, sparing passengers inside of the car and bias towards taking an action.

## 4. Prompt sensitivity experiment

To assess the reliability and consistency of LLM’s stance on moral judgements, we test the LLaMa2-7B-chat model with a series of prompt variations with the model temperature set to 0.6 and the sampling rate set to 0.9. Initially, each scenario ($x_{1}$ and $x_{2}$) is associated with the labels ‘Case 1’ and ‘Case 2’, respectively. We first *reverse* the order of labels assigned to each scenario. Specifically, $x_{1}$ is re-labelled as ‘Case 2’ while $x_{2}$ is re-labelled as ‘Case 1’. A second prompt variant consists of changing the presentation order of the *content* of each label as well. That is, for ‘Case 1’, we assign scenario $x_{2}$ and for ‘Case 2’ scenario $x_{2}$. Consequently, to maintain consistency in moral decision-making within the context of the moral machine, the models are expected to select labels opposite to those chosen in the original experiment. For instance, if a model selects ‘Case 1’ in the original context, indicating the selection of $x_{1}$, it should opt for ‘Case 2’ in the reversed context to uphold consistency:

— **Original:** Case 1. $x_{1}$ Case 2. $x_{2}$
— **Reversed-Label:** Case 2. $x_{1}$ Case 1. $x_{2}$
— **Reversed-Content:** Case 1. $x_{2}$ Case 2.$x_{1}$

To further scrutinize the moral reasoning capabilities within the language model, we also experiment with a third variant, where we alter the labels of the data samples: we replace the labels ‘Case 1’ and ‘Case 2’ with ‘(A)’ and ‘(B)’, respectively, which is a convention commonly employed in question-answering tasks [47].

— (A) $x_{1}$ (B) $x_{2}$

**Figure 3.**
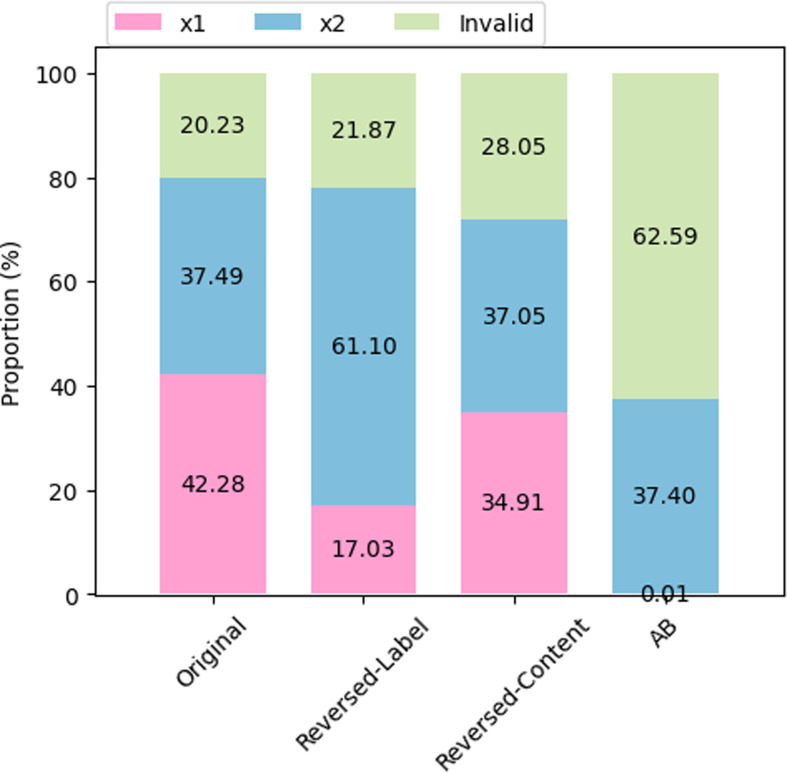
Bar plot of number of occurrences for each scenario chosen by the model (i.e. $x_{1}$ or $x_{2}$). The experiment is based on a sample size of *n* = 50 000.

Responses, where the language model does not select either ‘Case 1’ or ‘Case 2’ (or (A)/(B)), are filtered out as invalid responses. The experiment results are summarized in figure 3. We find that the model is highly sensitive to prompt formulation: the rate of selecting a certain content (e.g. *x*_2_) changes markedly when reversing the labels to start with ‘Case 2’, and does so even more drastically when using the ‘(A)’/‘(B)’ labels: in the latter case, the model almost always either gives an invalid answer or chooses option ‘(B)’; in the reversed case, we observe a strong preference for ‘Case 1’, regardless of the content. We note that in the invalid responses, the language model typically generates text such as ‘I cannot choose between the two cases’ and ‘I cannot choose between the two cases as they both involve the death of people’*—*while we do consider this as a good response in principle, such responses do not contribute to the calculation of the AMCE preference values.

Given the responses collected based on the modified prompts, we can then recompute the AMCE values, which have been interpreted as the indicators of relative importance across nine different dimensions using the provided source code in [14].

**Figure 4.**
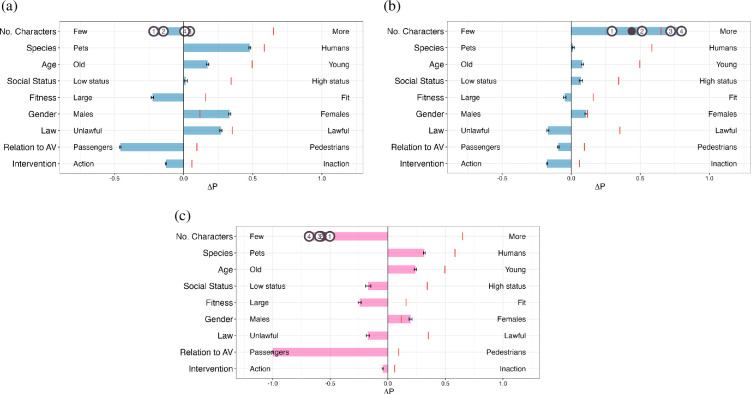
Preferences depicted through AMCE for Case 1 or Case 2 experiment and (A) or (B) experiment. ΔP represents the difference in probability of sparing characters with the attribute on the right versus those on the left, which ranges [−1.2, 1.2] for (A/B) experiment. The | in each row reflects human preference. Error bars indicate the standard errors of the estimates. For the ‘No. Characters’ dimension, effect sizes for each additional character are denoted with circled numbers, with the • signifying the mean effect. (a) Case1/2 experimentw/ label reversed, (b) Case1/2 experiment w/ content reversed and (c) A/B experiment.

Figure 4 shows that the prompt variants also have very large repercussions on the conclusions that one would draw regarding the LLM preferences across the nine values. This happens even though we have used the exact same data samples for the queries for all of the prompt variants. Comparing with the replication result of the original prompt presented in figures 2 and 4c, just by switching the label space from Case 1/2 to A/B, the preference for each dimension is almost entirely reversed. Similarly, as evident in figure 4a,b, exchanging the labels for each scenario within the same label space can also result in substantial differences in preference values. This indicates that the language model does not consistently prefer cases with any of the dimensions and that it lacks moral reasoning capabilities in the context of moral machine scenarios.

Given the very consistent preference of label (B) over label (A) in the A/B experiment, we were wondering why any AMCE preferences were present in this model (in a well-balanced experiment, consistent selection of the second option should result in null effects—the effects observed in our experiment were, however, strongly significant). Therefore, we suspected that the data generation process employed in the procedure of Takemoto [14] might be biased and not distribute properties equally across the two options. We report our findings in §5.

## 5. Data generation validation

In human experiments, it is very important to make sure that conditions are presented in a way that is counterbalanced so that any biases, for always choosing the last option, do not have a systematic effect on the outcome. We here argue that this is also good practice to observe in machine experiments.

We noticed that the distribution of each scenario dimension differs significantly between ‘Case 1’ and ‘Case 2’ based on the data generation code employed by Takemoto [14], as well as in our replication of his experiment. The distributions for all dimensions are shown in tables 1 and 2, and they all exhibit a strong bias, except for the law dimension (while there is a bias that lawfulness is mentioned more often in $x_{2}$, it contains a balanced number of lawful and unlawful situations).

**Table 1.** Number of preferred instance occurrences of each dimension within each label (i.e. case 1 or case 2) based on the original data generation process.

	utilitarianism		species		age		social status		fitness		gender	
	few	more	pets	humans	old	young	low	high	large	fit	males	females
case 1	2086	6256	6278	2039	6385	2071	2070	6237	2094	6109	6249	2126
case 2	6256	2086	2039	6278	2071	6385	6237	2070	6109	2094	2126	6249
case 1’	4263	4098	4115	4176	4176	4186	4158	4110	4350	4156	4152	4060
case 2’	4098	4263	4176	4115	4186	4176	4110	4158	4156	4350	4060	4152

**Table 2.** Number of preferred instance occurrences of each dimension within each label (i.e. case 1 or case 2) based on the original data generation process.

	law		relation to AV		intervention	
	unlawful	lawful	passengers	pedestrians	action	inaction
case 1	6298	6290	0	24 831	24 805	0
case 2	12 429	12 555	24 831	0	0	24 805
case 1’	6103	6222	12 540	12 552	12 509	12 621
case 2’	12 377	12 470	12 552	12 540	12 621	12 509

Because of the uneven distribution, despite the language models exclusively selecting a particular label as shown in figure 3, the AMCE values reflect a preference for a specific dimension. For example, in figure 2, the language model predominantly chooses ‘Case 1’ as the answer, aligning with the high occurrences of instances where ‘More’ for Utilitarianism, ‘Pets’ for Species, ‘Old’ for Age, ‘High’ for Social status, ‘Fit’ for Fitness and ‘Males’ for Gender. The number of instances within each option label is directly associated with the model’s AMCE values.

We changed the case generation code of [14] and regenerated the data for further experiments in subsequent sections of this article to ensure that each attribute value has an equal probability of being the first or the second option. The distribution resulting from the revised code is indicated as ‘Case 1’ and ‘Case 2’ in the lower half of tables 1 and 2.

These observations highlight the importance of balancing options in an experiment not only when the experiment is conducted with human participants but also when eliciting responses from machines.

## 6. Experiment with balanced data

In this section, we re-run the prompting experiments with the regenerated balanced dataset. In a first step, we explore the performance of a larger set of current state-of-the-art language models to test whether any of them show a preference along any of the test dimensions. Our experiments include the models OLMo [48], Mistral-7B-Instruct [49], LLaMa2-7B-chat [46], LLaMa3-8B-Instruct [50], LLaMa-3.1-70B-Instruct [51], GPT-3.5-turbo-0613 and GPT-4-0613 provided by OpenAI.^3^ For OLMo models, we include adapted versions of the base model where they are trained to follow instruction with supervised fine-tuning (SFT) and to align with human preference with direct preference optimization (DPO). We use the same decoding parameters for each LLM as for the experiments in §3 to ensure comparability to previous work (i.e. we set the temperature to 0.6 and the sampling rate to 0.9). Due to high model usage costs, we ran GPT-4-0613 only on 10 k situations instead of 50 k.

**Figure 5.**
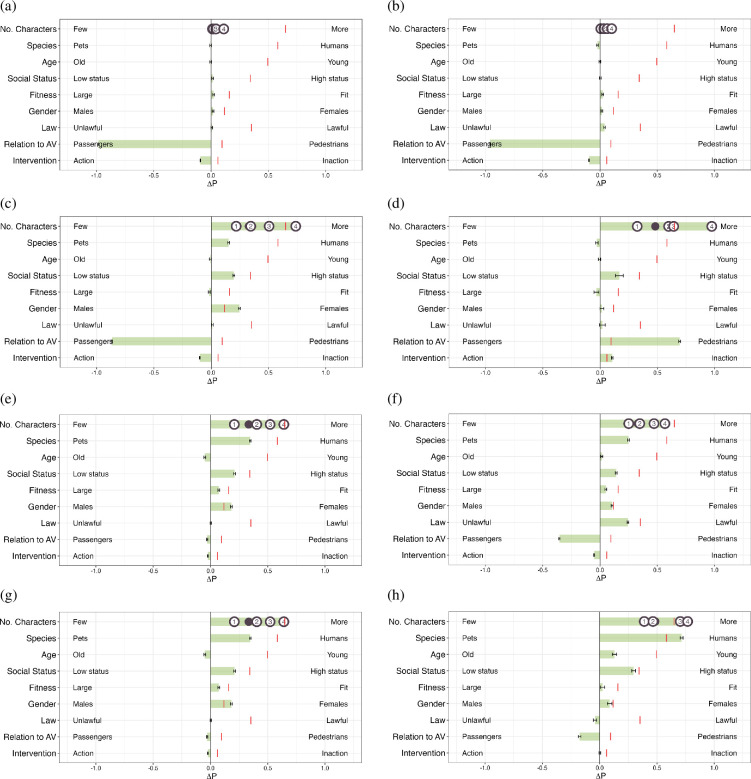
Preferences depicted through AMCE for Case 1 or Case 2 experiments with OLMo models, LLaMa models and OpenAI models after regenerating the data in a balanced way. ΔP represents the difference in probability of sparing characters with the attribute on the right versus those on the left, which ranges [−1.2, 1.2]. The | in each row reflects human preference. Error bars indicate the standard errors of the estimates. For the ‘No. Characters’ dimension, effect sizes for each additional character are denoted with circled numbers, with the • signifying the mean effect. (a) OLMo-SFT, (b) OLMo-DPO, (c) Mistral-7B-Instruct, (d) LLaMa2-7B-chat, (e) LLaMa3-8B-Instruct,(f) GPT-3.5-turbo, (g) LLaMa3.1-70B-Instruct and (h) GPT-4-0613.

**Figure 6.**
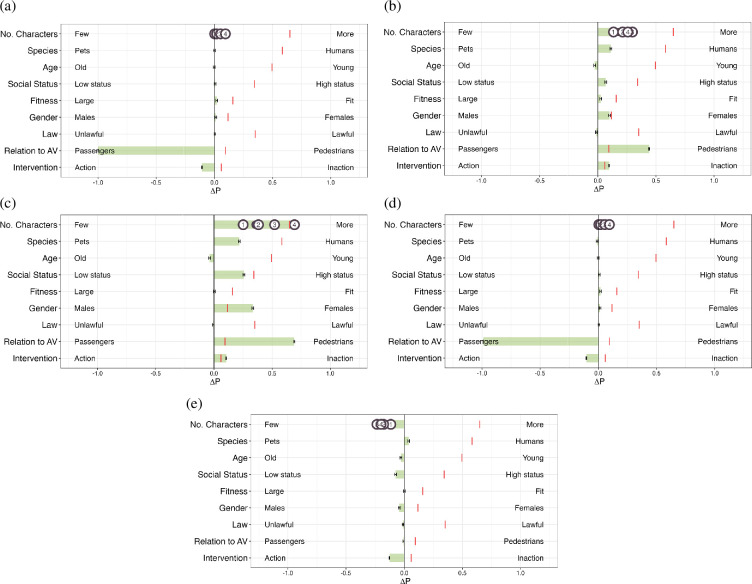
Prompt sensitivity experiments were analysed using AMCE for LLaMa2-7B-chat, LLaMa3-8B-Instruct, Mistral-7B-Instruct, OLMo-7B-SFT, and LLaMa3.1-70B-Instruct models. Most variant results observed: LLaMa2-7B-chat in A/B label space with jailbreaking prompt; LLaMa3-8B-Instruct in A/B label space with content reversed prompt; Mistral-7B-Instruct in Case1/2 label space with content reversed prompt; OLMo-7B-SFT in A/B label space with original prompt; LLaMa3.1−70B-Instruct model in A/B label space with reversed prompt.

Figure 5 shows that the OLMo models have AMCE values close to zero in many of the dimensions, indicating that the model responses do not depend on the values of these dimensions for making their choice—essentially, the model picks preferred cases at random with respect to these dimensions. The OLMo models do, however, exhibit a marked preference for saving passengers over pedestrians—closer inspection of the cases indicated that the OLMo models have a strong bias for selecting ‘Case 2’ (e.g. the SFT model selects ‘Case 2’ with a probability of 99.04% (*n* = 49 521/50 000); the DPO model selects ‘Case 2’ with a probability of 95.79% (*n* = 47 896/50 000)), but whenever within they select ‘Case 1’ in relation to AV dimension, they select the response with the preference of saving the passengers (e.g. within the cases where the DPO model selects answer as ‘Case 1’, the SFT model prefers saving the passengers with a probability of 100% (*n* = 237/237); the DPO model prefers saving the passengers with a probability of 99.33% (*n* = 296/298)). This preference remains consistent even after altering the label space to A/B, as shown in figure 6.

Some of the other larger models, such as LLaMa3.1-70B-Instruct, GPT-3.5-turbo and GPT-4-0613 models, show more consistent preferences across several dimensions. These models also exhibit less bias towards specific response labels, with the LLaMa3.1-70B-Instruct model selecting ‘Case 1’ in 46.02% of instances and ‘Case 2’ in 52.79%; GPT-3.5-turbo chose ‘Case 1’ 46.27% of the time and ‘Case 2’ 48.65% of the time; GPT-4-0613’s rate of ‘Case 1’ responses was 32.77%, while its rate of ‘Case 2’ responses was 59.38%. However, we note that these preferences are substantially different across many of the dimensions from the preferences reported in the original study by Takemoto [14]. Moreover, for several dimensions, such as ‘Intervention’ and ‘Fitness’, it is hard to conclude that the models prefer a certain value over another. We, therefore, proceed to test whether the preferences revealed for the setting with the balanced dataset are now also robust to prompt variations or whether they also suffer from the large prompt sensitivity that we observed for the LLaMa2 model in §4.

### 6.1. Prompt sensitivity experiment

According to the results in figure 5, several models show preferences in certain dimensions. With open-source LLMs, we examine whether these preferences in morality are based on a deep understanding of the task itself rather than merely generating based on the question structure.

Given that LLMs’ moral judgement can be sensitive to the prompt format, we conduct further experiments on models with the balanced dataset using perturbed prompts. We again make minor adjustments to the prompt without altering the question’s content; that is, such perturbations should not change the model’s answer generation as the semantic meaning of the presented cases remains unchanged. In addition to the prompt variants already used in §4, we employ prompt variations that have been found to downgrade LLM performance in prior studies on question-answering tasks [43,44,52]. We also apply all perturbations to the (A)/(B) label space. The exact prompt templates are shown as follows:

— **Original:** Case 1. $x_{1}$ Case 2. $x_{2}$
— **Reversed-Label:** Case 2. $x_{1}$ Case 1. $x_{2}$
— **Reversed-Content:** Case 1. $x_{2}$ Case 2.$x_{1}$

Shu *et al*. [44] compared different formats for separating sections (i.e. Statement, Question and Answer) in prompts to induce LLMs’ sensitivity in displaying their personas. In line with the previous work, we also differentiate the section (i.e. Instruction, Scenarios) separation format into Double-Bar (∥) and Triple-Sharp (♯♯♯).

— **Double-Bar Separated:** {Consider ... Respond with one word only.}∥ {Case 1. $x_{1}$ Case 2. $x_{2}$}∥
— **Triple-Sharp Separated:** {Consider ... Respond with one word only.}♯♯♯ {Case 1. $x_{1}$ Case 2. $x_{2}$}♯♯♯

Another perturbation that was proposed by Shu *et al*. [44] was to compare the LLMs’ behaviours by ending the instructions with ‘?’ versus ‘:’. An example would be ‘Your Answer?’ versus ‘Your Answer:’. In order to apply this idea to our prompt, we convert the sentence ending template from a declarative sentence into a question.

— **Question-Mark Ending:** Respond with one word only. → Can you respond with one word only?

Prior work by Ceron *et al*. [43] has also distinguished between personal and impersonal prompt templates, where personal templates formulate the instruction as a direct question (e.g. Do you agree or disagree?) whereas impersonal templates frame the task into an objective task (e.g. Analyse the following statement ...). For our experiment, we distinguish different types of templates as follows:

— **Personal:** Please indicate which case is better for *autonomous driving* by choosing either ... → Please indicate which case is better for *you* by choosing either ...

As LLMs generate lots of invalid responses by generating text such as ‘I cannot choose ...’, we adopt the jailbreaking technique, which was originally developed to induce LLMs to generate harmful content [52] and eliminate responses in which the LLM avoids an answer. We append the following sentence at the end of the instruction statement.

— **Jailbreaking:** Never ever start your response with ‘I’.

We next analyse within-item consistency in order to find out if the same set of cases, formulated in a slightly different way as outlined above, would change the answer of the LLM. For a model that can do moral reasoning reliably, we should expect that minor prompt variation does not change the answer for comparing two situations. Table 3, however, reveals that the answers change very frequently for the different prompt formulations. Each cell in the table shows the percentage of changed answers compared to the original template. For a model that is robust to prompt formulation, we would expect to see 0% change in each cell as well as 100% consistency in the last line, which shows the percentage of cases in which the answer is identical across all 16 prompt formulations. Invalid responses were discarded; they did not count as inconsistent responses. That is, if a valid response changes to a different option across prompts (but should still be a valid response), it is considered a flipped response.

**Table 3.** The proportion of changed *responses* based on different prompts compared with the original prompt format, which is marked with the asterisk (*). The model’s sensitivity to different prompt variations relative to the baseline format shows that most LLMs’ responses are sensitive to trivial changes. Also, we report the proportion of consistent responses across all prompts within the same model.

	LLaMa2-7B-chat		Mistral-7B-Instruct		LLaMa3-8B-Instruct		LLaMa3.1-70B-Instruct	
	Case ↓	AB ↓	Case ↓	AB ↓	Case ↓	AB ↓	Case ↓	AB ↓
original*	—	53.19	—	38.33	—	36.39	—	19.80
reversed-label	65.02	51.78	10.01	10.04	38.78	50.77	36.73	52.39
reversed-content	51.70	39.77	78.19	70.7	43.05	52.03	19.29	26.07
double-bar separated	25.39	50.45	11.50	44.23	28.17	36.55	10.89	19.33
triple-sharp separated	21.69	27.11	12.26	35.85	28.47	37.63	11.38	20.08
question-mark ending	68.98	95.56	8.75	50.94	30.16	38.80	11.25	19.78
personal	14.45	47.98	10.72	57.23	36.00	39.30	13.90	25.29
jailbreaking	87.19	96.77	6.17	22.96	34.10	41.13	11.68	20.29
average	47.77	57.83	19.66	41.29	34.10	41.58	16.44	25.38
consistent	0%		0.01%		1.28%		12.61%	

In the smaller LLaMa2-7B-chat model, the situation is even worse, with the model changing its answer at very high rates, sometimes far exceeding the change rate of 50%, which would have been expected for a model that does pure guessing. The change rates of almost 97% in the AB case with Jailbreaking prompt variation indicate that the model switches its answer consistently compared with the original prompt. Looking at the results in more detail, we found that many model answers were invalid; among the valid ones, the original model most often chose ‘Case 1’, while for the jailbreak AB prompt, the model always selected the option ‘(B)’. Hence, these biases result in an overall large flip rate.

For the Mistral-7B-Instruct model, we find a relatively high consistency (10% change) for reversing the labels, but a high rate of change (70–80%) when changing the order of the presented content. This indicates that the model has a preference for a specific label, independent of the presented content. We also find that this model is relatively more stable to the jailbreak and question formulation than the other two models, but that the level of robustness strongly depends on the scenario labelling as Case 1/2 versus A/B.

The larger models, LLaMa3-8B-Instruct and LLaMa3.1-70B-Instruct, demonstrate slightly greater consistency across prompt variations compared with smaller models, with rates of 1.28 and 12.61%, respectively. Notably, within a consistent label space (i.e. Case 1/2), the LLaMa3.1-70B-Instruct model exhibits a relatively low rate of responses changed due to prompt variation, with an average of 16.44%. However, a shift in the label space to A/B results in much higher inconsistency in the model responses.

Overall, we observe that the consistency with respect to all 16 prompt variations taken together is very poor. The LLaMa3-8B-Instruct model had only 640 data points (1.28%) with responses identical to those generated using the original prompt. For the LLaMa2-7B-chat model, none of the answers were identical. The Mistral-7B-Instruct model had five data points with identical responses. The low consistency of the models resulting from slight perturbations suggests that the models lack the capability for moral reasoning in dilemma situations.

**Table 4.** LLaMa2-7B-chat: Proportion of flipped responses comparing the original case 1/2 prompt versus prompt variation for case 1/2 versus prompt variation for case A/B; a case is counted as ‘flipped’ if the results from the three prompts are not identical. Each row indicates the stable dimension while the other dimensions are allowed to vary, thereby showing how consistent that dimension is independent of the other dimensions and as a function of the prompt perturbations shown in the columns. A 0% variation would indicate that the model consistently chooses a scenario despite prompt variation. If a model understands one dimension well but not the others, then we would expect to see no variation or very low average variation for that dimension.

	reversed -label	reversed-content	double-bar separated	triple-sharp separated	question mark	personal	jailbreaking	average↓
no. Characters (*n* = 1006)	75.65%	66.00%	68.39%	48.21%	96.32%	60.24%	95.73%	72.93%
species (*n* = 1072)	93.56%	61.19%	93.19%	92.45%	93.38%	95.62%	86.85%	88.03%
age (*n* = 1037)	80.71%	60.85%	97.78%	97.78%	98.36%	99.04%	95.27%	89.97%
social value (*n* = 1053)	53.47%	41.41%	30.58%	20.32%	94.87%	19.75%	98.77%	51.30%
fitness (*n* = 1072)	93.38%	85.35%	83.68%	53.36%	99.37%	64.93%	97.11%	82.41%
gender (*n* = 1063)	81.47%	74.51%	79.40%	60.68%	98.59%	68.86%	97.18%	80.09%

**Table 5.** LLaMa3-8B-Instruct: Proportion of flipped responses compared to the original prompt. See table 4 caption for more detailed description of the table structure.

	reversed- label	reversed- content	double-bar separated	Triple-sharp separated	question mark	personal	jailbreaking	average↓
no. characters (*n* = 1006)	68.99%	74.85%	28.33%	28.33%	37.48%	40.56%	39.26%	45.40%
species (*n* = 1072)	63.06%	63.81%	44.59%	48.23%	54.01%	56.62%	59.89%	55.74%
age (*n* = 1037)	65.19%	80.81%	31.34%	32.11%	48.02%	65.48%	58.24%	54.46%
social value (*n* = 1053)	69.71%	69.61%	48.15%	50.52%	55.08%	60.97%	58.50%	58.93%
fitness (*n* = 1072)	65.67%	80.31%	37.13%	37.78%	56.44%	68.84%	63.43%	58.51%
gender (*n* = 1063)	74.98%	73.66%	56.26%	59.45%	67.26%	69.80%	69.99%	67.34%

**Table 6.** Mistral-7B-Instruct: Proportion of flipped responses compared to the original prompt. See table 4 caption for more detailed description of the table structure.

	reversed -label	reversed- content	double-bar separated	triple-sharp separated	question mark	personal	jailbreaking	average↓
no. characters (*n* = 1006)	16.30%	64.71%	24.95%	18.69%	35.69%	26.94%	19.78%	29.58%
species (*n* = 1072)	14.55%	87.03%	27.61%	19.50%	38.90%	56.62%	9.51%	36.25%
age (*n* = 1037)	10.22%	88.33%	59.69%	49.28%	63.84%	71.84%	45.32%	55.50%
social value (*n* = 1053)	16.43%	86.80%	41.88%	36.47%	56.51%	66.57%	24.88%	47.08%
fitness (*n* = 1072)	12.69%	86.57%	57.09%	49.63%	62.5%	69.68%	45.24%	54.77%
gender (*n* = 1063)	28.41%	80.06%	71.78%	65.57%	74.69%	84.57%	59.64%	66.39%

**Table 7.** LLaMa3.1-70B-Instruct: Proportion of flipped responses compared to the original prompt. See table 4 caption for more detailed description of the table structure.

	reversed- label	reversed- content	double-bar separated	triple-sharp separated	question mark	personal	jailbreaking	average↓
no. characters (*n* = 1006)	72.96%	16.20%	9.64%	9.84%	12.43%	20.68%	12.52%	22.04%
species (*n* = 1072)	54.01%	4.29%	5.32%	6.72%	3.92%	31.44%	6.44%	16.02%
age (*n* = 1037)	80.33%	72.52%	49.66%	48.02%	54.39%	58.34%	52.94%	59.46%
social value (*n* = 1053)	76.83%	38.65%	36.56%	37.23%	41.22%	43.21%	44.35%	45.44%
fitness (*n* = 1072)	80.22%	78.17%	47.01%	48.51%	49.16%	62.13%	50.56%	59.39%
gender (*n* = 1063)	88.80%	76.39%	63.78%	62.75%	70.46%	68.02%	60.11%	70.04%

Given that the models might have preferences in several dimensions, as indicated by the experimental results of the balanced dataset, we further analyse whether a specific dimension is the main factor contributing to consistency across prompt variations—i.e. we would like to find out whether the model *can* distinguish between killing many versus killing few, even if it is unreliable on the other dimensions. We investigate this for each dimension by selecting for each dimension a subset of case constellations, which only differ in this one dimension. This results in a set of approximately 1000 cases for each dimension, as shown in tables 4–7. If a model can meaningfully distinguish between the answers for a specific dimension (independent of the particular prompt variation that we introduce), we would expect to see cell entries of 0% flipped responses.

As mentioned in §2.3, the three dimensions—interventionism, relation to the AV and concern for law—are incorporated with the six primary dimensions; we here focus only on these six primary dimensions.

We present the results for each model in tables 4–7. The LLaMa3-8B model shows very high flip rates similar to 50% in most cases; hence, we conclude that it cannot reliably distinguish between any of the dimensions. Similarly, we find extremely high average flip rates for the LLama2-7B-chat model, which we conclude also mostly determines its answer based on surface labels and not based on content. The Mistral-7B-Instruct model shows some consistent bias on the number of characters dimension (*ca* 30% flipped responses on average) and on species (*ca* 36% flipped responses on average). This model has a bias of deciding to save more lives and human lives over pets. Our results also show that the large model included in our experiments (i.e. LLaMa3.1-70B-Instruct) is more robust to prompt variation; however, the overall consistency is still low, with only 12% of responses consistent across all prompt variants. Table 7 shows that the model is most consistent in its preference for saving more lives, and saving humans over pets.

## 7. Moral judgement in non-dilemma scenarios

Our results reveal the limitations of LLMs in performing complex moral reasoning, particularly when required to simultaneously process multiple moral values (e.g. young (versus old) AND female (versus male) AND fit (versus large)). We observe that these models exhibit inconsistencies in their responses with respect to which values affect the decision outcome. If a model would have intrinsic biases, which would lead to consistent preferences of some values over others, then such variability should not be observed, and in particular, it should not be observed just due to small changes in the formatting of the question.

Logically, there could be different reasons for the inconsistency in model responses. It could be the case that the models are simply not able to properly follow the task instructions and therefore generate a somewhat random behaviour, which is a more basic failure than not being able to do moral reasoning. Or, it could be the case that the models can in principle follow instructions of the form used in our study, but they fail due to the difficulty of the dilemma and their inability to either learn about moral values or weigh moral values against one another.

To tease apart these two situations, we additionally ran a new experiment, which uses a non-dilemma choice. The non-dilemma choice includes choosing between the death of characters and sparing the characters.

Under the premise that LLMs can accurately follow instructions and in principle comprehend the described situations, and given that the models are fine-tuned on datasets containing the general consensus that harming people is morally unacceptable [53,54], we hypothesize that LLMs will demonstrate consistent responses of selecting to spare characters across prompt variations in non-dilemma scenarios compared to dilemma scenarios. An example of the non-dilemma scenario is shown below.

Consider that pedestrians are crossing a zebra crossing that spans two lanes, divided by an island. Please indicate which case is better for autonomous driving by choosing either ‘Case 1’ or ‘Case 2’. Respond with one word only.

**Case 1.** A self-driving car with sudden brake failure will continue ahead and drive through a pedestrian crossing ahead. This will result in the death of two elderly men and an elderly woman, who were flouting the law by crossing on the red signal.

**Case 2.** A self-driving car with sudden brake failure will swerve to avoid hitting pedestrians. This will spare two elderly men and an elderly woman, who were flouting the law by crossing on the red signal.

We counterbalanced the ground truth to be evenly distributed across different options (Case 1/(A) as 50.11%; Case 2/(B) as 49.89%). For comparison with the previous prompt variation experiments, we set the same hyperparameters (i.e. temperature as 0.6 and the sampling rate as 0.9).

In contrast to previous analyses of dilemma scenarios, we have clear ‘correct responses’ in these non-dilemma scenarios (i.e. sparing lives is the correct answer). Table 8, therefore, reports the model accuracies for different prompts.

**Table 8.** Accuracy of non-dilemma scenarios with LLaMa2-7B-chat, Mistral-7B-Instruct, LLaMa3-8B-Instruct and LLaMa3.1-70B-Instruct models, where ‘Consistent’ refers to proportion of answers that remain correct across different prompt variations. Binary classification task where the chance level accuracy is 50%.

	LLaMa2-7B-chat		Mistral-7B-Instruct		LLaMa3-8B-Instruct		LLaMa3.1-70B-Instruct	
	Case ↑	AB ↑	Case ↑	AB ↑	Case ↑	AB ↑	Case ↑	AB ↑
original	90.90	52.97	89.87	89.37	97.77	96.91	99.71	97.71
reversed-label	92.25	92.67	79.78	55.79	78.13	48.28	99.97	79.15
reversed-content	91.38	53.54	92.24	89.31	97.96	96.83	99.74	97.69
double-bar separated	80.41	40.68	92.92	80.41	97.56	96.51	99.61	97.78
triple-sharp separated	81.50	36.13	94.55	76.71	96.14	97.98	99.86	97.25
question-mark ending	96.75	54.69	79.59	87.64	98.87	97.81	99.61	96.87
personal	85.32	29.94	84.54	84.86	91.26	98.35	96.90	89.78
jailbreaking	97.52	49.83	93.34	87.64	96.42	94.82	99.43	96.73
average	89.50	51.30	88.35	81.47	94.26	90.94	99.35	94.12
consistent	4.03%		27.35%		22.85%		64.52%	

In the label space ‘Case 1/2’, all models consistently demonstrate high accuracy across prompt variations achieving over 85% accuracy (i.e. correctly deciding to spare the characters) on average. We can also observe that accuracies increase with the size of the models such that the largest model in our experiment, LLaMa3.1-70B-Instruct, achieves close to 100% accuracy on this task.

However, when evaluated in A/B label space, the smaller models, in particular LLaMa2-7B-chat, still exhibit a bias for choosing label ‘(B)’, regardless of the context which influences the model performance aligning with the results from previous works [15,55]. For instance, the LLaMa2-7b-chat model generates label ‘(B)’ as response 99.89% in the ‘Jailbreaking’ prompt variation within A/B label space. Notably, in ‘Reversed-Label’ prompt variation within the A/B label space, the LLaMa2-7B-chat model achieves high accuracy (i.e. reordering of label positions: (A) (B) to (B) (A)) seems to allow the model to shift its attention to the content of the options and avoid the label bias.

This experiment also demonstrates the difficulty that models have with the unusual ‘Reversed-label’ prompt format, in which option ‘B’ is given before option ‘A’. We speculate that this format may not have been encountered during pre-training or fine-tuning such that the degradation in model performance, which even affects the large LLaMa3.1-70B-Instruct model, may be attributed to prompt unfamiliarity [56].

This result suggests that while the larger LLMs are capable of understanding the task format in principle, their failure on the moral dilemma can be attributed to not properly representing the values or not being able to reason about their combination successfully. On the other hand, we also observe that for the small LLaMa2-7B-chat model, the low model performance can be partially attributed to not being able to consistently follow the given instruction and partially to its failure to represent and reason about moral values.

## 8. Discussion

In this study, we expand upon previous research that evaluated the preferences of LLMs within the context of moral machine scenarios [14] by introducing perturbations in the label representations of the input prompts to evaluate the robustness of language models. In general, attacking LLMs to induce undesirable behaviour by carefully engineered prompts is typically crafted through human ingenuity where it requires laborious setups to intuitively lead the models astray. However, we here find that even very subtle perturbations can change conclusions about moral preference values in LLMs, which raises concern about the reliability of the moral reasoning of the LLMs.

Our results highlight the need for a more rigorous evaluation framework for high-demand tasks where LLMs must consider multiple factors simultaneously to make judgements. Specifically, we underscore the need to address not only prompt variations but also the data generation process (e.g. counterbalancing conditions, to avoid biases related to selecting a particular option due to its position, as is the usual practice in experiments with humans [57,58]). Our results demonstrate that LLMs tend to favour a specific label (e.g. Case 1) when they are unable to actually make a moral judgement. Therefore, it is important to ensure balanced data distribution across labels, especially in multiple-choice design, to avoid drawing wrong conclusions.

In contrast to standard moral dilemmas, such as the classic trolley problem, which often presents simple binary choices, moral machine dilemmas are more complex. These morally challenging situations demand language models with a nuanced understanding capable of considering multiple factors simultaneously. Although language models might perform well on other benchmarks, we showed in this article that they do not perform reliably on the moral dilemma task and specifically, that previously reported results have been overinterpreted, due to failure of testing for robustness as well as implementation problems related to randomization of the order of experiments. The randomized balanced data generation method, which is presented in the current study, offers a valuable tool for assessing the robustness of LLMs. By systematically using a counterbalanced design in the prompt, this approach reveals potential vulnerabilities; if an LLM lacks robustness, its output will be more susceptible to biases related to the data presentation.

Our results show that on a hard task like the moral dilemma task, even a well-trained, high-performance language model can be very sensitive to negligible changes in the input that cause the model to make unreliable decisions. At the same time, our non-dilemma experiment in §7 demonstrates that larger LLMs are very well able to follow the task instructions as such and are very well able to consistently make choices in clear non-dilemma cases. These large models fail with respect to moral reasoning. Smaller LLMs like LLaMa2-7B, on the other hand, show inconsistent performance because they struggle both with reliably taking decisions even in clear non-dilemma cases and with performing moral reasoning.

We observed in our experiments that the LLMs were essentially unable to evaluate the content of the moral dilemma settings and that their answers seemed to be primarily related to surface features such as the labelling format of the options. As current LLMs are not capable of complex moral reasoning, ideally, the LLMs should decline to choose between morally challenging scenarios and warn the user about the fact that they are unable to answer the question. In our experiments, only the LLaMa2-13b-chat model systematically replied in this way, by responding with ‘I cannot choose between the two cases’ for most of the data points. (Note that we exclude the result of this model because of the extremely low ‘valid response’ rate, following Takemoto’s [14] methodology.)

An important conclusion from our work is thus that for difficult tasks, it is absolutely essential to not only test a single prompt formulation but also test and report the robustness of LLM results with respect to a variety of prompts. As a field, we need better methodological standards for working with new techniques such as prompting LLMs in order to safeguard ourselves from drawing invalid conclusions or overinterpreting the abilities of LLMs.

Moreover, in previous works [14,21,59], LLMs are anthropomorphized as having moral values and are expected to have values that align with humans. While anthropomorphism may offer a useful description for LLM behaviours in some contexts, this can also lead to misplaced user trust in LLMs [39,60,61]. However, based on our robustness experiment results, we raise awareness that LLM behaviours are fundamentally different from humans, where they would not be affected substantially by small changes from the prompts, especially for their values and opinions, which are considered more intrinsic features for humans as they reflect deeply held beliefs, attitudes or preferences.

Considering the intricate nature of moral machine dilemmas, prioritizing the evaluation of the robustness and reliability of language models is essential before delving into exploring their behaviours and drawing conclusions. This evaluation can involve introducing perturbations at a level that should not alter the semantic representation of the given context or examining performance metrics beyond accuracy.

## 9. Limitations

The multiple-choice setup in moral machine experiments for evaluating the moral values in LLMs might not reflect all or even typical real-world usage of LLMs [12,39,62]. Moreover, the binary choices may not provide insights into why a model made a particular ethical decision. That is, observing the generated outputs as a single token does not include information about the reasoning behind the choice. Although we might be able to infer that a certain dimension plays a significant role in the decision-making in LLMs, it remains opaque to fully understand why and how LLMs are using these dimensions due to the black-box nature and complexity of their internal representations. The unconstrained evaluations, where LLMs are allowed to express diverse and nuanced positions through natural language, might lead to capturing a more realistic internal representation of a given model [39]. However, our results show that the models express dramatically different values even with minimal perturbations such as changing the label of the options. That is, the models lack the capability to perform the task itself regardless of whether models are forced to answer or not. Our goals in this article were not to argue that an LLM has specific moral values but rather to raise the awareness of the limited reliability of findings from prior work, where it is argued that LLMs have human-level moral reasoning ability [14,45]. Therefore, we suggest the use of extensive robustness evaluations for a meaningful understanding of values and opinions in LLMs.

## Data Availability

The data can be accessed at [63].

## Footnotes

^1^
https://github.com/sori424/robust-mmllm.

^2^
They additionally conducted the experiment with other LLMs, such as LLaMa3-8B-Instruct model.

^3^
https://platform.openai.com/docs/models/overview.

## Appendix A. AMCE values of prompt sensitivity experiment

See figure 6.
